# Bioalerts: a python library for the derivation of structural alerts from bioactivity and toxicity data sets

**DOI:** 10.1186/s13321-016-0125-7

**Published:** 2016-03-04

**Authors:** Isidro Cortes-Ciriano

**Affiliations:** Unité de Bioinformatique Structurale, CNRS UMR 3825, Département de Biologie Structurale et Chimie, Institut Pasteur, 25, rue du Dr. Roux, 75015 Paris, France

**Keywords:** Structural alerts, Circular fingerprints, Morgan fingerprints, Toxicology, Bioactivity

## Abstract

**Background:**

Assessing compound toxicity at early stages of the drug discovery process is a crucial task to dismiss drug candidates likely to fail in clinical trials. Screening drug candidates against structural alerts, i.e. chemical fragments associated to a toxicological response prior or after being metabolized (bioactivation), has proved a valuable approach for this task. During the last decades, diverse algorithms have been proposed for the automatic derivation of structural alerts from categorical toxicity data sets.

**Results and conclusions:**

Here, the python library *bioalerts* is presented, which comprises functionalities for the automatic derivation of structural alerts from categorical (dichotomous), e.g. toxic/non-toxic, and continuous bioactivity data sets, e.g. $K_{i}$ or $\hbox {pIC}_{50}$ values. The library *bioalerts* relies on the RDKit implementation of the circular Morgan fingerprint algorithm to compute chemical substructures, which are derived by considering radial atom neighbourhoods of increasing bond radius. In addition to the derivation of structural alerts, *bioalerts* provides functionalities for the calculation of unhashed (keyed) Morgan fingerprints, which can be used in predictive bioactivity modelling with the advantage of allowing for a chemically meaningful deconvolution of the chemical space. Finally, *bioalerts* provides functionalities for the easy visualization of the derived structural alerts.

**Electronic supplementary material:**

The online version of this article (doi:10.1186/s13321-016-0125-7) contains supplementary material, which is available to authorized users.

## Background

The early assessment of compound toxicity is crucial in drug discovery to dismiss drug candidates displaying undesirable safety profiles. This reduces the risk of investing time and resources on compounds likely to fail at clinical trials. Computational toxicology aims at predicting *in silico* the health and environmental risks associated to compound exposure or intake. The main benefits of these approaches are the potential reduction of the usage of animal models and their low cost in comparison to in vitro and in vivo methods.

The derivation of structural alerts, i.e. chemical fragments associated to a toxicological response prior or after being metabolized (bioactivation), is an area of intense development in toxicology. A plethora of studies in the last decades have proved the suitability of screening drug candidates against structural alerts known to be implicated in compound toxicity [1–4]. In silico approaches to catalogue, rationalize and identify structural alerts are generally divided into two groups. The first category comprises knowledge-based expert systems [5–8]. These methods gather and annotate structural alerts derived through human experience or from the scientific literature. These data can be used to define sets of rules for the evaluation of compound toxicity. Nonetheless, further expanding knowledge-based expert systems, i.e. adding more structural alerts by toxicology experts or from curation of scientific literature, is an arduous and cost-inefficient task. In addition, the need for human subjective intervention might lead to divergent perceptions about the toxicity of particular structural alerts.

The second category is composed of data-driven systems [9]. In this case, machine learning and data mining techniques are applied to large toxicity data sets to identify structural alerts in an automatic and unbiased fashion. This second class of methods differ with respect to knowledge-based expert systems in that (1) they are fast, exhaustive, deterministic, and automatic, and (2) there is no need for human intervention for the derivation of the strucutural alerts, although it might be required for their interpretation. Diverse notions underlie the data-driven algorithms reported in the literature, such as maximum common substructure (MCS) calculation [10], emerging pattern mining [9, 11], or clique-based techniques [12]. The functionalities of these methods have been exposed and compared on categorical toxicity data sets, such as mutagenicity or carcinogenicity. However, the notion of identifying chemical fragments that enrich for a biological endpoint, be it toxicity or in vitro enzyme inhibition, can be extended to data sets reporting continuous compound activity values.

Here, the python library *bioalerts* is presented, which comprises functionalities for the automatic derivation of structural alerts from categorical (dichotomous), e.g. toxic/non-toxic, and continuous bioactivity data sets, e.g. $K_{i}$ or $\hbox {pIC}_{50}$ values. The workflow proposed by Ahlberg et al. [13] is followed for the derivation of structural alerts from categorical data—with the exception that to generate compound substructures circular Morgan fingerprints are used instead of a signature descriptor [14, 15]—whereas the pipeline published by Cortes-Ciriano et al. [16] is followed when using continuous bioactivity data. Ahlberg and coworkers showed that their pipeline leads to comparable results to both manual derivation of structural alerts and a clique-based method, namely PAFI [17]. However, the computational efficiency of their method was proved to be significantly higher; in fact, training and prediction times were reduced by up to three orders of magnitude in comparison to PAFI [13]. The library *bioalerts* relies on the RDKit implementation of the circular Morgan fingerprint algorithm to compute chemical substructures, which are derived by considering radial atom neighbourhoods of increasing bond radius. Morgan fingerprints were chosen given the high retrieval rates obtained with them in comparative virtual screening studies [18, 19]. Thus, they appear to efficiently account for chemical aspects related to bioactivity. In addition to the derivation of structural alerts, *bioalerts* provides functionalities for the calculation of unhashed (keyed) Morgan fingerprints, which can be used in predictive bioactivity modelling with the advantage of allowing for a chemically meaningful deconvolution of the chemical space [16]. Finally, *bioalerts* provides functionalities for the easy visualization of the derived structural alerts.

## Implementation

The library *bioalerts* is implemented in python. It is mainly based on the RDKit implementation of circular Morgan fingerprints, licensed under a 3-clause BSD license [20], and depends on the following python modules: numpy, operator, os, pandas, scipy, and sys [21–23]. The library is divided in three modules, namely: (1) *LoadMolecules*, (2) *Alerts*, and (3) *FPCalculator*. These modules allow to read molecule files, compute hashed and unhashed Morgan fingerprints in binary and count format, and to derive structural alerts from continuous and categorical bioactivity data sets. The implementation of the three modules is serial. Their functionalities are presented in the following subsections.

### Reading molecule files

Molecule files can be read with the method *ReadMolecules()* from the class *bioalerts.LoadMolecules.LoadMolecules()*. The available input file formats are: (1) SMILES, (2) SDF, and (3) mol2. Once the molecules are loaded into a python list, the method *extract_substructure_information()* from the class *bioalerts.LoadMolecules.GetDataSetInfo()* permits the generation of a dictionary of substructures, whose keys correspond to substructure unambiguous integer identifiers, and the values to the molecule indices within the molecule list. Only the substructures with a bond radius allowed by the user through the argument *radii* are considered to build the substructure dictionary. This dictionary, which defines in terms of substructure composition the input set of molecules (e.g. training set), serves to compute unhashed Morgan fingerprints and to derive structural alerts.

### Computation of Morgan fingerprints

Morgan fingerprints encode chemical structures by considering atom neighbourhoods [24]. For their computation, each substructure in a molecule, with a maximal user-defined bond radius, is assigned an unambiguous integer identifier. These identifiers can be mapped into an unhashed or hashed array. For the hashed array, the position in the array where the substructures are mapped is given by the modulo of the division of the substructure identifier by the fingerprint size. In the case of unhashed (keyed) fingerprints, each bit in the fingerprint is associated to only one substructure. Thus, the length of the unhashed fingerprints is equal to the number of distinct substructures present in the molecule set. Morgan fingerprints can be generated as binary, recording the presence or absence of each substructure, or count format, recording the number of occurrences of each substructure in a given compound.

The methods *calculate_hashed_fps()* and *calculate_unhashed_fps()* from the class *bioalerts.FPCalculator.CalculateFPs()* permit the computation of hashed and unhashed Morgan fingeprints in both count and binary format, respectively. Both methods rely on the RDKit function *rdkit.Chem.AllChem.GetMorganFingerprintAsBitVect()*. Since the positions of the substructures in the unhashed fingerprints depend on the training set, the method *calculate_unhashed_fps()* allows the computation of unhashed fingerprints for new compounds using a basis defined by the substructures present in a training set. This basis is defined by the keys of the substructure dictionary calculated for the molecules from the training set. Images of the substructures highlighted within the molecules where they appear can be saved to individual Portable Document Format (PDF) files. To allow further interpretation of the unhashed fingerprints, the execution of the method *calculate_unhashed_fps()* generates a dictionary, namely *CalculateFPs.substructures_smiles*, with the substructure integer idenfiers as keys, and their structure in SMILES format as values. The usage of the class *CalculateFPs()* and its methods is illustrated in the tutorial and documentation provided in the Supplementary Information (Additional files 1 and 2).

**Fig. 1:**
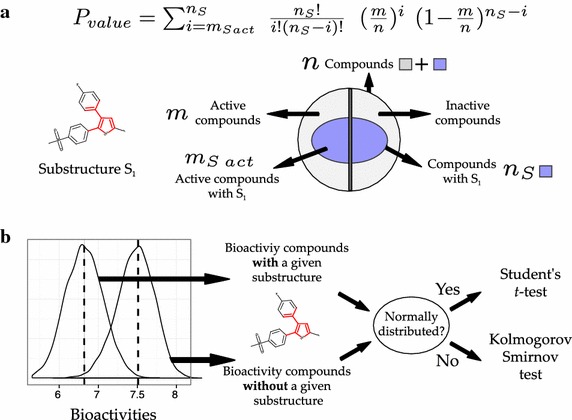
Illustration of the pipelines proposed for the derivation of structural alerts using categorical (**a**) and continuous (**b**) data

### Derivation of structural alerts from categorial bioactivity data sets

The derivation of structural alerts from dichotomous bioactivity data follows the work by Ahlberg et al. [13]. The probability for a substructure to be a structural alert is derived from the probability density function of the binomial distribution (Eq. 1). The question to be answered here is (Fig. 1a): which is the probability of having observed by chance $m_{S\ act}$ active compounds (or more) with a given substructure ($S_1$), given that the substructure is present in $n_s$ compounds in a training set of *n* compounds where *m* are active (Fig. 1a)?

A high probability (i.e. *P* value) indicates that it is likely to obtain by chance $m_{S\ act}$ active compounds with substructure $S_1$ from the binomial distribution defined by the training data, and thus, substructure $S_1$ is not likely to be correlated to compound activity.

By contrast, a low *P* value indicates that it is not likely to have observed by chance $m_S\ act$ active compounds with substructure $S_1$ from the underlying binomial distribution defined by the training data. Thus, the presence of substructure $S_1$ is significantly associated to compound activity.

The *P* values are calculated from:

1 $$\begin{aligned} P_{value} = \sum _{i=m_{S\ act}}^{n_{S}} \frac{n_{S}!}{i! (n_{S}-i)!} \ \left( \frac{m}{n}\right) ^{i} \ \left( 1-\frac{m}{n}\right) ^{n_{S}-i } \end{aligned}$$

where *n* is the total number of compounds in the training set, *m* the number of active compounds, $n_s$ the number of compounds with a given substructure $S_1$, and $m_S\ act$ the total number of compounds that are active and present substructure $S_1$.

A training set is used to derive a substructure dictionary. This dictionary serves to calculate the *P* value for the substructures present in the molecules from a test or external set with the method *calculate_p_values()* from the class *bioalerts.Alerts()*. This method calculates the substructures for the external molecules and their associated *P* value. The method *calculate_p_values()* requires three parameters to be defined, namely: (1) *threshold_nb_substructures*: the threshold for the number of substructures (default value equal to 5) [13], (2) *threshold_pvalue*: *P* value threshold (default value equal to 0.05), and (3) *threshold_frequency*: substructure frequency threshold. The threshold for the number of substructures, *threshold_nb_substructures*, indicates the minimum number of compounds in the training set with a given substructure ($n_s$) required to proceed to the calculation of the *P* value for that substructure. For instance, if the thresehold is set to 5 [13], and only 4 compounds from the training set present a given substructure $S_1$, the algorithm will not calculate the *P* value for $S_1$. On the other hand, the *P* value threshold, *threshold_pvalue*, indicates the level of significance ($\alpha$) to consider a given substructure as structural alert. Finally, the substructure frequency threshold, *threshold_frequency*, corresponds to the ratio $m_{S\ act}$/$n_s$. Therefore, if there are too few active molecules with a given substructure $S_1$ with respect to the total number of molecules with that substructure, the *P* value for $S_1$ will not be computed.

Overall, the structure of the algorithm is as follows:

- Take as input a series of molecules (i.e. training set) and derive a dictionary of substructures (reference set). Only the substructures with a bond radius within the set of bond radii specified by the user will be considered.
- For each molecule from an external (or test) set, do:
  - For each set of substructures rooted at a given heavy atom, Set$_{\mathrm{substr.}}$, do:
    1. Among the substructures in this set that have not been processed yet, select the substructure *S* of smallest radius
    2. If substructure *S* is in the reference substructure set derived from the training set, continue
    3. If the number of compounds with substructure S in the training set is higher than the value of the argument *threshold_nb_substructures*, continue
    4. If the ratio $m_{S\ act}$/$n_s$ is higher than the value of the argument *threshold_frequency*, continue
    5. Compute *P* value for substructure *S*
    6. If the *P* value is below the *P* value threshold, *threshold_pvalue*, label substructure *S* as significant. In this case, substructure *S* will not be considered even if it appears in other molecules from the external molecule set yet not processed. In addition, substructures comprising substructure *S* (i.e. substructures of higher bond radius rooted at the same heavy atom present in Set$_{\mathrm{substr.}}$ but not yet processed) will not be considered in future iterations.

To control the familywise error rate the Bonferroni correction can be applied to the computed *P* values by setting the argument *Bonferroni* of the method *calculate_p_values()* to True (default). The Bonferroni correction consists of multiplying the *P* values by the total number of substructures for which *P* values were computed. Alternatively, the significance level can be divided by the number of computed *P* values. In that case, the significance level would be compared to the computed *P* values without transforming them.

### Derivation of structural alerts from continuous bioactivity data sets

The module *calculate_p_values()* from the class *bioalerts.Alerts.CalculatePvaluesContinuous()* permits the derivation of structural alerts from data sets reporting continuous compound activity values, e.g. $\hbox {pIC}_{50}$. To identify which substructures from a test molecule significantly contribute to bioactivity on the biomolecular system under study, two bioactivity distributions are defined (Fig. 1b), namely: (1) distribution *A*, comprising the bioactivity values for those compounds from the training set presenting a given substructure, and (2) distribution *B*, comprising the bioactivity values for those compounds not presenting that substructure in the same training set. The normality of these distributions is assessed with the Shapiro–Wilk test ($\alpha = 0.05$). If both *A* and *B* are normally distributed, a two-tailed *t* test for independent samples is applied to statistically evaluate the difference between these two distributions. By contrast, if *A* and *B* do not follow a normal distribution, the Kolmogorov Smirnov test is used instead. If the difference between *A* and *B* is significant, the considered substructure is assumed to have an influence on bioactivity. The sign of the difference between the mean value of *A* and *B* indicates whether the presence of that substructure increases or decreases compound activity on the studied biological system.

The method *calculate_p_values()* requires two parameters. The first one, *threshold_nb_substructures*, indicates the minimum number of compounds in the training set presenting a given substructure that is required to compute the significance (*P* value) for that substructure. The second one, *threshold_ratio*, refers to the ratio between the number of compounds from the training set with a substructure and the size of the training set. If this ratio is below the value set for the parameter *threshold_ratio* the algorithm will not further consider that substructure.

Overall, the structure of the algorithm is as follows:

- Take as input a series of molecules (i.e. training set) and derive a dictionary of substructures (reference set). Only the substructures with a bond radius within the set of bond radii specified by the user will be considered.
- For each substructure, *S*, in the molecules from an external (or test) set, do:
  - If substructure S is in the reference substructure set derived from the training set and has not been previously processed, continue
  - If the number of compounds with substructure S in the training set is higher than the value of the argument *threshold_nb_substructures*, continue
  - If the ratio between the number of compounds with substructure *S* from the training set and the total number of distinct compounds from the training set is higher than the value set for the argument *threshold_ratio*, continue
  - Compute *P* value for substructure *S* using the Student’s *t* test or the Kolmogorov Smirnov test.

The substructures identified with the algorithm along with additional information, e.g. the associated *P* values and the bioactivity difference between distributions *A* and *B* (effect size), are saved to a pandas data frame [22], which can be further saved to a .xlsx file for further processing and visualization (Additional files 1 and 2). As in the previous case, the Bonferroni correction can be applied to the computed *P* values if the argument *Bonferroni* of the method *calculate_p_values()* is set to True (default).

## Discussion and conclusion

An open source implementation of two methodologies for the automatic derivation of structural alerts from bioactivity data sets is presented. Additionally, the library *bioalerts* permits the computation of unhashed (keyed) Morgan fingerprints. The performance of unhashed and hashed Morgan fingerprints has been shown to be comparable on continuous bioactivity data sets [16] (Additional file 2). Nonetheless, building predictive models with unhashed fingeprints enables the deconvolution of the models in a chemically meaningful way [16, 25, 26], thus increasing model interpretability. The functionalities of *bioalerts* are illustrated in a tutorial (Additional file 2) using three diverse bioactivity data sets, namely: (1) Ames mutagenicity data for 1752 compounds [27], (2) $\hbox {pIC}_{50}$ values for 2311 compounds on human cyclooxygenase (COX) 2 [16], and (3) blood–brain barrier (BBB) data for 157 organic compounds [28].

Although the method for the derivation of structural alerts from continuous bioactivity data has been recently validated on a human cyclooxygenases data set [16], it is of paramount importance to bear in mind the following limitations. This method treats the effect of indivudal substructures on compound activity as independent events. However, it was shown by Klekota et al. [29] that the contribution to biological activity of some substructures depends on the presence of others. Similarly, it is important to note a second scenario where none of the methods would be suited. We can envision a data set where all active compounds present a given substructure $S_{1}$ not implicated in the studied biological response, and substructure $S_{2}$ enriching for activity, wheras all inactive compounds in that data set do not present neither $S_{1}$ nor $S_{2}$. The presented approaches would identify both substructures $S_{1}$ and $S_{2}$ as implicated in bioactivity, whereas only $S_{2}$ actually is. Given that in practice the last situation might not be common when modelling large data sets, it is advisable to use large and chemically diverse training sets.

Finally, another important consideration when deriving structural alerts from continuous bioactivity data is the effect size required to privilege or dismiss compounds presenting a given substructure. The effect size corresponds to the difference between the two distributions from which the statistical test, e.g. *t* test, is calculated. For instance, (1) distribution *A* comprising the bioactivities for all compounds in a data set harboring a given substructure, and (2) distribution *B* comprising the bioactivities for the remaining compounds. In some cases, depending on the sample sizes, a highly significant *P* value might be obtained for a small size effect, e.g. a tenth of a $\hbox {pIC}_{50}$ unit. From a medicinal chemistry standpoint that difference might be negligible [30]. Therefore, it is paramount to pay especial attention to the effect size in addition to the *P* values.

Overall, *bioalerts* constitutes an open source python library for the derivation of structural alerts from categorical and continuous data sets using two methodologies that have been previously validated. In addition, *bioalerts* functionalities include the computation of unhashed Morgan fingerprints, which can be further used in e.g. predictive bioactivity modelling, clustering or similarity searching.

## Availability and requirements

- **Project name:** bioalerts
- **Project home page:** Source code is available at http://github.com/isidroc/bioalerts. Users of *bioalerts* are encouraged to visit this site for future versions and improvements.
- **Operating system(s):** Platform independent
- **Programming language:** Python
- **License:** GNU GPL version 3
- **Any restrictions to use by non-academics:** none.

## Additional files

10.1186/s13321-016-0125-7 Bioalerts library and documentation. The file *bioalerts.zip* expands to a folder containing the library scripts and documentation. The folder *build* contains an HyperText Markup Language (HTML) tree which documents the library *bioalerts* using reStructuredText (.rst) as markdown language and processed with sphinx (www.http://sphinx-doc.org/). The documentation can be browsed by opening the file *index.html* file in any HTML browser. The documentation of the python library *RDKit* can be accessed at www.rdkit.org.
10.1186/s13321-016-0125-7 Bioalerts Tutorial (*tutorial.zip*). The tutorial is provided in three file formats: (i) file *bioalerts_tutorial.html*, which can be browsed in any HTML browser, (ii) file *bioalerts_tutorial*, and (iii) file *bioalerts_tutorial.ipynb*, which can be opened with ipython notebook. This tutorial illustrates the functionalities of *bioalerts* on three real world data sets available at http://github.com/isidroc/bioalerts.
