# Supplementary material for: Bioalerts: a python library for the derivation of structural alerts from bioactivity and toxicity data sets
Source: J Cheminform. 2016 Mar 4;8:13. doi: 10.1186/s13321-016-0125-7 (PMC4779235; doi:10.1186/s13321-016-0125-7)
Supplement: Supplementary file 1 — 10.1186/s13321-016-0125-7 Bioalerts library and documentation. The file bioalerts.zip expands to a folder containing the library scripts and documentation. The folder build contains an HyperText Markup Language (HTML) tree which documents the library bioalerts using reStructuredText (.rst) as markdown language and processed with sphinx (www.http://sphinx-doc.org/). The documentation can be browsed by opening the file index.html file in any HTML browser. The documentation of the python library RDKit can be accessed at www.rdkit.org. [file 13321_2016_125_MOESM1_ESM.zip › bioalerts/build/Alerts.html]

Alerts: Derivation of structural alerts — bioalerts 1.0 documentation


### Navigation

- index
- next |
- previous |
- bioalerts 1.0 documentation »

# Alerts: Derivation of structural alerts¶

This module is composed of the following three classes:

- GetSubstructuresFromReferenceDataset
- CalculatePvaluesCategorical
- CalculatePvaluesContinuous

## CalculatePvaluesCategorical¶

*class* CalculatePvaluesCategorical(*max\_radius*)¶
:   |  |  |
    | --- | --- |
    | Variables: | - **max\_radius** (*int*) – maximum substructure radius considered. - **output** – pandas dataframe containing the following columns: (i) ‘Compound ID’, (ii) ‘p\_value’, (iii) ‘Compounds with substr.’, (iv) ‘activity label’, (v) ‘Substructure’, (vi) ‘Substructure in Molecule’, (vii) ‘Comp. with substr. active’, and (viii) ‘Comp. with substr. inactive’.   :vartype output: pandas.core.frame.DataFrame |

    calculate\_p\_values(*mols*, *substructure\_dictionary*, *bioactivities*, *mols\_ids*, *threshold\_nb\_substructures*, *threshold\_pvalue*, *threshold\_frequency*, *active\_label=1*, *inactive\_label=0*)¶
    :   |  |  |
        | --- | --- |
        | Parameters: | - **mols** (*list*) – list of molecules from the test set in rdkit mol format (rdkit.Chem.rdchem.Mol) - **substructure\_dictionary** (*dict*) – dictionary of substructures derived from the training set molecules that will be used as a reference. This dictionary can be calculated with the method bioalerts.GetDataSetInfo.extract\_substructure\_information() - **bioactivities** (*list*) – activity labels for the molecules from the training set. - **mols\_ids** (*list*) – ids of the molecules input through the parameter mols. - **threshold\_nb\_substructures** (*int*) – minimum number of substructures (n’) required to compute P values - **threshold\_pvalue** (*float*) – significance level - **threshold\_frequency** (*float*) – the lower threshold for the ratio m’ / n’ (see manuscript for details) - **active\_label** – the label for active molecules, e.g. “Active” or 1 - **inactive\_label** – the label for inactive molecules - **Bonferroni** (*Boolean*) – whether Bonferroni correction is to be applied to the computed P values. If set to True, only the substructures passing this correction will be reported in the field output. |

    HTMLOutputWriter(*self*, *output\_filename*)¶
    :   |  |  |
        | --- | --- |
        | Parameters: | **output\_filename** (*str*) – name of the html file to which the data frame ‘output’ will be saved. |

    XlSXOutputWriter(*self,frame, output\_filename, molCol=['Substructure',"Substructure in Molecule"], size=(300,300)*)¶
    :   |  |  |
        | --- | --- |
        | Parameters: | - **frame** – data frame to be written to the xlsx file. Generally, this will be the data frame in the field output of the class CalculatePvaluesContinuous(). - **output\_filename** (*str*) – name of the xlx file to which the data frame will be saved - **molCol** (*list*) – columns of the data frame output that will be saved to the xlsx file - **size** (*tuple*) – size of the substructure and compound images |

## CalculatePvaluesContinuous¶

*class* CalculatePvaluesContinuous(*radii\_ext*)¶
:   |  |  |
    | --- | --- |
    | Variables: | - **radii\_ext** (*list*) – substructure radii to be considered - **output** – pandas dataframe containing the following columns: (i) ‘Compound ID’, (ii) ‘Number compounds’, (iii) ‘statistic’, (iv) ‘p\_value’, (v) ‘Diff. distribution means (w - wo)’, (vi) ‘Compounds with substr.’, (vii) ‘Substructure’, and (viii) ‘Substructure in Molecule’. |

    calculate\_p\_values(*mols*, *substructure\_dictionary*, *bioactivities*, *mols\_ids*, *threshold\_nb\_substructures*, *threshold\_pvalue*, *threshold\_ratio*, *Bonferroni=True*)¶
    :   |  |  |
        | --- | --- |
        | Parameters: | - **mols** (*list*) – list of molecules from the test set in rdkit mol format (rdkit.Chem.rdchem.Mol) - **substructure\_dictionary** (*dict*) – dictionary of substructures derived from the training set molecules that will be used as a reference. This dictionary can be calculated with the method bioalerts.GetDataSetInfo.extract\_substructure\_information() - **bioactivities** (*list*) – activity values for the molecules from the training set. - **mols\_ids** (*list*) – ids of the molecules input through the parameter mols. - **threshold\_nb\_substructures** (*int*) – minimum number of substructures (n’) required to compute P values - **threshold\_pvalue** (*float*) – significance level - **threshold\_ratio** (*float*) – ratio between the number of compounds from the training set with a substructure and the size of the training set. - **Bonferroni** (*Boolean*) – whether Bonferroni correction is to be applied to the computed P values. If set to True, only the substructures passing this correction will be reported in the field output. |

    HTMLOutputWriter(*self*, *output\_filename*)¶
    :   |  |  |
        | --- | --- |
        | Parameters: | **output\_filename** (*str*) – name of the html file to which the data frame ‘output’ will be saved. |

    XlSXOutputWriter(*self,frame, output\_filename, molCol=['Substructure',"Substructure in Molecule"], size=(300,300)*)¶
    :   |  |  |
        | --- | --- |
        | Parameters: | - **frame** – data frame to be written to the xlsx file. Generally, this will be the data frame in the field output of the class CalculatePvaluesContinuous(). - **output\_filename** (*str*) – name of the xlx file to which the data frame will be saved - **molCol** (*list*) – columns of the data frame output that will be saved to the xlsx file - **size** (*tuple*) – size of the substructure and compound images |

### Table Of Contents

- Alerts: Derivation of structural alerts
  - CalculatePvaluesCategorical
  - CalculatePvaluesContinuous

#### Previous topic

LoadMolecules: Load Molecules

#### Next topic

FPCalculator: Fingerprint Calculator

### This Page

- Show Source

### Quick search


Enter search terms or a module, class or function name.

### Navigation

- index
- next |
- previous |
- bioalerts 1.0 documentation »

© Copyright 2015, Isidro Cortes Ciriano.
Created using Sphinx 1.2.3.
