# Supplementary material for: Bioalerts: a python library for the derivation of structural alerts from bioactivity and toxicity data sets
Source: J Cheminform. 2016 Mar 4;8:13. doi: 10.1186/s13321-016-0125-7 (PMC4779235; doi:10.1186/s13321-016-0125-7)
Supplement: Supplementary file 1 — 10.1186/s13321-016-0125-7 Bioalerts library and documentation. The file bioalerts.zip expands to a folder containing the library scripts and documentation. The folder build contains an HyperText Markup Language (HTML) tree which documents the library bioalerts using reStructuredText (.rst) as markdown language and processed with sphinx (www.http://sphinx-doc.org/). The documentation can be browsed by opening the file index.html file in any HTML browser. The documentation of the python library RDKit can be accessed at www.rdkit.org. [file 13321_2016_125_MOESM1_ESM.zip › bioalerts/build/FPCalculator.html]

FPCalculator: Fingerprint Calculator — bioalerts 1.0 documentation


### Navigation

- index
- previous |
- bioalerts 1.0 documentation »

# FPCalculator: Fingerprint Calculator¶

This module can be used to extract the substructures, of a user-defined radius,
from a list of molecules in rdkit Mol class format.

The module is composed of the following class:

- CalculateFPs

## CalculateFPs¶

*class* CalculateFPs(*radii*, *mols*, *reference\_substructure\_keys={}*)¶
:   *CalculateFPs* serve to calculate the following types of Morgan fingerpints:

    1. Hashed fingerprints in binary format
    2. Hashed fingerprints in count format
    3. Unhashed fingerprints in binary format
    4. Unhashed fingerprints in count format

    Morgan fingerprints encode chemical structures by considering atom neighbourhoods.
    Each substructure in the molecule set, with a maximal user-defined bond radius, is assigned an unambiguous integer identifier.
    These identifiers are mapped either into an unhashed or hashed array.
    For the hashed array, the position in the array where the substructures will be mapped is given by the modulo of the division
    of the substructure identifier by the fingerprint size.
    In the case of unhashed (keyed) fingerprints, each bit in the fingerprint is associated to only one substructure,
    producing a length of the unhashed fingerprints equal to the number of distinct substructures present in the dataset.
    Both hashed and unhashed fingerprints can be stored in binary and count format.
    In count format, each bit in the fingerprint accounts for the number of times each substructure is present in a given compound,
    whereas in binary format each bit encodes whether a substructure is present in a compound (1), irrespective of the number of occurrences, or not (0).

    |  |  |
    | --- | --- |
    | Variables: | - **radii** – radii of the substructures that will be used to generate the fingerprints for the molecules specified in the argument *mols*. - **max\_radius** – maximum substructure radius considered. - **mols** – input molecules for which fingerprints will be calculated. - **reference\_substructure\_keys** – dictionary of substructure identifiers that will be used to calculate the unhashed version of the Morgan fingerprints. This dictionary is calculated with the method *LoadMolecules.GetDataSetInfo.extract\_substructure\_information().* - **substructure\_dictionary** – dictionary containing substructure for the molecules specified in the argument *mols*, and for which fingerprints will be calculated. - **mols\_reference\_for\_unhashed** – reference set of molecules whose substructures are to be considered when computing unhashed fingerprints. This molecule set can be the same set of molecules for which the user wants to compute the fingerprints, *i.e.* *mols*, or a different molecule set. For instance, if a bioactivity model trained on a given data set using unhashed fingerprints is to be applied on an external data set, the fingerprints for the new molecules should be the same as those used to train the model. Thus, the refernce set of molecules (*mols\_reference\_for\_unhashed*) would correspond in this case to the molecules used to train the aforesaid model. |

    The value for the following attributes is set when running the fingerprint calculation methods explained below.

    |  |  |
    | --- | --- |
    | Variables: | - **columns\_unhashed** (*numpy.ndarray*) – fingerprint identifiers corresponding to the columns in the unhashed fingerprints. - **substructure\_ids** (*numpy.ndarray*) – identifiers for the substructures. - **fps\_hashed\_binary\_quick** (*numpy.ndarray*) – hashed fingerprints in binary format - **fps\_hashed\_binary** (*numpy.ndarray*) – hashed fingerprints in binary format. - **fps\_hashed\_counts** (*numpy.ndarray*) – unhashed fingerprints in count format. - **fps\_unhashed\_binary** (*numpy.ndarray*) – unhashed fingerprints in binary format. - **fps\_unhashed\_counts** (*numpy.ndarray*) – unhashed fingerprints in count format. - **substructures\_smiles** (*dict*) – dictionary containing the smiles for the substructures. |

    calculate\_hashed\_fps\_binary\_quick(*nBits*)¶
    :   Fast class method to compute hashed fingerprints in binary format. The computed hashed fingerprints are stored in *CalculateFPs.fps\_hashed\_binary\_quick*.

        |  |  |
        | --- | --- |
        | Parameters: | **nBits** (*int*) – fingerprint size |

    calculate\_hashed\_fps\_counts(*nBits*)¶
    :   Class method to compute hashed fingerprints in binary and count format. The computed fingerprints are stored in *CalculateFPs.fps\_hashed\_binary* and *CalculateFPs.fps\_hashed\_counts*, respectively.

        |  |  |
        | --- | --- |
        | Parameters: | **nBits** (*int*) – fingerprint size |

    calculate\_unhashed\_fps(*draw\_substructures=False*, *image\_directory='./images\_substructures'*)¶
    :   Class method to compute unhashed fingerprints in binary and count format. If *draw\_substructures* is set to True, depictions of each substructure in .pdf format, in the context of a molecules from *mols* where the substructure is present, will be saved to the directory specified in the argument *image\_directory*.

        |  |  |
        | --- | --- |
        | Parameters: | - **draw\_substructures** (*bool*) – if set to True, depictions for all substructures (with a bond radius allowed by the user through the argument radii) present in the training set of molecules will be generated. - **image\_directory** (*str*) – directory to save the substructure depictions |

### Table Of Contents

- FPCalculator: Fingerprint Calculator
  - CalculateFPs

#### Previous topic

Alerts: Derivation of structural alerts

### This Page

- Show Source

### Quick search


Enter search terms or a module, class or function name.

### Navigation

- index
- previous |
- bioalerts 1.0 documentation »

© Copyright 2015, Isidro Cortes Ciriano.
Created using Sphinx 1.2.3.
