# Supplementary material for: Bioalerts: a python library for the derivation of structural alerts from bioactivity and toxicity data sets
Source: J Cheminform. 2016 Mar 4;8:13. doi: 10.1186/s13321-016-0125-7 (PMC4779235; doi:10.1186/s13321-016-0125-7)
Supplement: Supplementary file 1 — 10.1186/s13321-016-0125-7 Bioalerts library and documentation. The file bioalerts.zip expands to a folder containing the library scripts and documentation. The folder build contains an HyperText Markup Language (HTML) tree which documents the library bioalerts using reStructuredText (.rst) as markdown language and processed with sphinx (www.http://sphinx-doc.org/). The documentation can be browsed by opening the file index.html file in any HTML browser. The documentation of the python library RDKit can be accessed at www.rdkit.org. [file 13321_2016_125_MOESM1_ESM.zip › bioalerts/build/genindex.html]

Index — bioalerts 1.0 documentation


### Navigation

- index
- bioalerts 1.0 documentation »

# Index

**C**
| **E**
| **G**
| **H**
| **L**
| **R**
| **X**

## C

|  |  |
| --- | --- |
| calculate\_hashed\_fps\_binary\_quick() (CalculateFPs method)  calculate\_hashed\_fps\_counts() (CalculateFPs method)  calculate\_p\_values() (CalculatePvaluesCategorical method)  (CalculatePvaluesContinuous method)  calculate\_unhashed\_fps() (CalculateFPs method) | CalculateFPs (built-in class)  CalculatePvaluesCategorical (built-in class)  CalculatePvaluesContinuous (built-in class) |

## E

|  |
| --- |
| extract\_substructure\_information() |

## G

|  |
| --- |
| GetDataSetInfo (built-in class) |

## H

|  |
| --- |
| HTMLOutputWriter() (CalculatePvaluesCategorical method)  (CalculatePvaluesContinuous method) |

## L

|  |
| --- |
| LoadMolecules (built-in class) |

## R

|  |
| --- |
| ReadMolecules() |

## X

|  |
| --- |
| XlSXOutputWriter() (CalculatePvaluesCategorical method)  (CalculatePvaluesContinuous method) |

### Quick search


Enter search terms or a module, class or function name.

### Navigation

- index
- bioalerts 1.0 documentation »

© Copyright 2015, Isidro Cortes Ciriano.
Created using Sphinx 1.2.3.
