# Supplementary material for: Bioalerts: a python library for the derivation of structural alerts from bioactivity and toxicity data sets
Source: J Cheminform. 2016 Mar 4;8:13. doi: 10.1186/s13321-016-0125-7 (PMC4779235; doi:10.1186/s13321-016-0125-7)
Supplement: Supplementary file 1 — 10.1186/s13321-016-0125-7 Bioalerts library and documentation. The file bioalerts.zip expands to a folder containing the library scripts and documentation. The folder build contains an HyperText Markup Language (HTML) tree which documents the library bioalerts using reStructuredText (.rst) as markdown language and processed with sphinx (www.http://sphinx-doc.org/). The documentation can be browsed by opening the file index.html file in any HTML browser. The documentation of the python library RDKit can be accessed at www.rdkit.org. [file 13321_2016_125_MOESM1_ESM.zip › bioalerts/build/LoadMolecules.html]

LoadMolecules: Load Molecules — bioalerts 1.0 documentation


### Navigation

- index
- next |
- previous |
- bioalerts 1.0 documentation »

# LoadMolecules: Load Molecules¶

This module serves to load molecules from smiles, sdf or mol2 files.

The module is composed of the classes:

- LoadMolecules
- GetDataSetInfo

## LoadMolecules¶

*class* LoadMolecules(*input\_file*, *verbose=True*, *delimiter="t"*, *name\_field="\_Name"*)¶

This class can be used to load molecules to python from files in smiles, sdf and mol2 format
using the rdkit library (www.rdkit.org).
Molecules are thus saved as RDkit Mol class objects.
It contains the following instance attributes:

|  |  |
| --- | --- |
| var input\_file: | input file containing the molecules. |
| vartype input\_file: | |
|  | str |
| var verbose: | print to stdout the information about the progress of the calculations if set to True (default). |
| vartype input\_file: | |
|  | bool |
| var delimiter: | delimiter between fields in the input molecule file. Used with smiles files. |
| vartype input\_file: | |
|  | str |
| var name\_field: | field in sdf files containing that will be used to get the molecule names. |
| vartype name\_field: | |
|  | str |

The value for the following attributes are set when calling the method LoadMolecules.ReadMolecules():

|  |  |
| --- | --- |
| var mols: | molecules that were properly processed. |
| vartype mols: | list |
| var molserr: | indices for the input molecules (starting at zero) that were incorrectly processed. This indices are also written to the file “incorrect\_molecules.csv”. |
| vartype molserr: | |
|  | list |
| var nb\_mols: | number of molecules properly processed. |
| vartype nb\_mols: | |
|  | int |
| var mols\_ids: | indices of the molcules properly processed with respect to their position in the input file. |
| vartype mols\_ids: | |
|  | list |
| var name\_field: | name of the field corresponding to the molecule names in the input file. |
| vartype name\_field: | |
|  | str |

ReadMolecules(*titleLine=False*, *smilesColumn=0*, *nameColumn=1*)¶

Method to read the molecules contained in the input file (LoadMolecules.input\_file).
The arguments of this method are only used when reading molecules from smiles files.

| param titleLine: | |
| --- | --- |
|  | From the RDkit documentation: “If true, the first line is assumed to list the names of properties in order seperated by ‘delimiter’”. Here, ‘delimiter’ corresponds to LoadMolecules.delimiter. |
| type titleLine: | bool |
| param smilesColumn: | |
|  | column (starting at zero) containing the smiles in the input file. |
| type smilesColumn: | |
|  | int |
| param nameColumn: | |
|  | column (starting at zero) containing the molecule names in the input file. Note that (from the rdkit doc): “If the input file has a title line and more than two columns (smiles and id), the additional columns will be used to set properties on each molecule. The properties are accessible using the mol.GetProp(propName) method.” |
| type nameColumn: | |
|  | int |

## GetDataSetInfo¶

*class* GetDataSetInfo(*name\_field=None*)¶

This class can be used to load molecules in smiles sdf and mol2 format.

|  |  |
| --- | --- |
| var name\_field: | name of the data field containing the name of the molecules. Although the default value is None (*bool*), the name of the field if set when instantiating the class would be a string (*str*). |
| vartype name\_field: | |
|  | str |
| var nb\_substructures: | |
|  | total number of substructures, with a radius comprised in the argument *radii* of *GetDataSetInfo.extract\_substructure\_information()*, from the molecules specified in the argument *mols* of the method *GetDataSetInfo.extract\_substructure\_information()* (see below). |
| vartype nb\_substructures: | |
|  | int |
| var max\_radius: | maximum substructure radius considered. This corresponds to the maximum value of the argument *radii* of *GetDataSetInfo.extract\_substructure\_information()*. |
| vartype max\_radius: | |
|  | int |
| var substructure\_dictionary: | |
|  | dictionary containing the substructures, with a radius comprised in the argument *radii* of *GetDataSetInfo.extract\_substructure\_information()*, and the molecules where they appear. Keys correspond to molecules names, whereas values correspond to |
| vartype substructure\_dictionary: | |
|  | dict |

*GetDataSetInfo* contains the following methods:

extract\_substructure\_information(*radii*, *mols*)¶
:   This method extracts the substructures from the molecules (argument *mols*)

whose radius is comprised in the argument *radii*.
Each substructure in the molecule set (*mols*) is assigned an unambiguous integer identifier,
which are kept in *GetDataSetInfo.substructure\_dictionary*.
The information about the substructures is kept in the fields indicated above.

|  |  |
| --- | --- |
| param radii: | substructure radii to be considered |
| param mols: | molecules from which the substructures are to be extracted. |
| type radii: | list |
| type mols: | list |

### Table Of Contents

- LoadMolecules: Load Molecules
  - LoadMolecules
  - GetDataSetInfo

#### Previous topic

Welcome to bioalerts’s documentation!

#### Next topic

Alerts: Derivation of structural alerts

### This Page

- Show Source

### Quick search


Enter search terms or a module, class or function name.

### Navigation

- index
- next |
- previous |
- bioalerts 1.0 documentation »

© Copyright 2015, Isidro Cortes Ciriano.
Created using Sphinx 1.2.3.
