# Supplementary material for: Bioalerts: a python library for the derivation of structural alerts from bioactivity and toxicity data sets
Source: J Cheminform. 2016 Mar 4;8:13. doi: 10.1186/s13321-016-0125-7 (PMC4779235; doi:10.1186/s13321-016-0125-7)
Supplement: Supplementary file 2 — 10.1186/s13321-016-0125-7 Bioalerts Tutorial (tutorial.zip). The tutorial is provided in three file formats: (i) file bioalerts_tutorial.html, which can be browsed in any HTML browser, (ii) file bioalerts_tutorial, and (iii) file bioalerts_tutorial.ipynb, which can be opened with ipython notebook. This tutorial illustrates the functionalities of bioalerts on three real world data sets available at http://github.com/isidroc/bioalerts. [file 13321_2016_125_MOESM2_ESM.zip › tutorial/bioalerts_tutorial.html]

bioalerts\_tutorial


# bioalerts Tutorial¶

### **bioalerts: A Python Package for the Derivation of Structural Alerts from Bioactivity Data Sets**¶

Isidro Cortés Ciriano (isidrolauscher@gmail.com). 2015.

This tutorial illustrates the functionaties provided by the three modules (*LoadMolecules, Alerts, FPCalculator*) comprised in the package library **bioalerts**.

Firstly, import **bioalerts** and other packages required for this tutorial.

Note: do not forget to add the path to your python envirnonment variable, e.g.:
export PYTHONPATH=$PYTHONPATH:/usr/local/share/RDKit:/Users/user1/Desktop/bioalerts/

In [1]:

```
import bioalerts
import numpy as np
```

In [2]:

```
from bioalerts import LoadMolecules, Alerts, FPCalculator
```

In [3]:

```
from rdkit.Chem.Draw import IPythonConsole
from rdkit.Chem import PandasTools
# Machine learning modules
import sklearn
from sklearn import preprocessing
import scipy
from sklearn.metrics import mean_squared_error
from sklearn.ensemble import RandomForestRegressor
from sklearn.metrics import r2_score
```

This tutorial was run with the following python and module versions:

In [4]:

```
import sys, numpy as np, scipy as sc, rdkit, matplotlib as pylab, pandas as pd, IPython
print " Python:", sys.version, "\n"
print " Numpy:", np.__version__
print " Scipy:", sc.__version__
print " Rdkit:", rdkit.rdBase.rdkitVersion
print " Matplotlib:", pylab.__version__
print " Pandas:", pd.__version__
print " Ipython:", IPython.__version__
print " Scikit-Learn:", sklearn.__version__
print " Scipy:", scipy.__version__
```

```
 Python: 2.7.2 (default, Oct 11 2012, 20:14:37) 
[GCC 4.2.1 Compatible Apple Clang 4.0 (tags/Apple/clang-418.0.60)] 

 Numpy: 1.9.2
 Scipy: 0.16.0
 Rdkit: 2014.09.2
 Matplotlib: 1.4.3
 Pandas: 0.15.2
 Ipython: 3.0.0
 Scikit-Learn: 0.16.1
 Scipy: 0.16.0
```

In [5]:

```
import time
initial_time = time.clock()
```

# The following three data sets will be used in this tutorial:¶

1. Derivation of Structural Alerts from categorical bioactivity data:

   **Ames mutagenicity data**:
   1 indicates Ames positive (i.e. causes mutations in bacteria), and 0 Ames negative.
   Extracted from Young et al. Chemometrics and Intelligent Laboratory Systems 60 (2002) 5–11.

1. Derivation of Structural Alerts from continuous bioactivity data:

   **Human cyclooxygenase (COX) 2**:
   Compound activity is encoded with pIC50 values.
   Extracted from Cortés-Ciriano et al. Journal of Cheminformatics 2015, 7:1.
2. Calculation of Morgan hashed and unhashed fingerprints and their application in predictive bioactivity modelling:

   **Blood-brain barrier (BBB)**:
   Blood–brain barrier permeability for organic compounds.
   Extracted from Zhang et al. Pharmaceutical Research 2008, Vol. 25, No. 8.

# A. Reading molecules and extracting their substructures¶

### A.1 Class to read the molecules:¶

```
bioalerts.LoadMolecules
```

In [6]:

```
molecules = bioalerts.LoadMolecules.LoadMolecules("./datasets/Young_2002.sdf",
                                                  verbose=False)
molecules.ReadMolecules()
print "Total number of input molecules: ", len(molecules.mols)
```

```
Format of the structures file = SDF
266 molecules (starting at zero) could not be processed.

This information has been saved in the following file: incorrect_molecules.csv

NOTE: the indexes of the molecules start at zero. Thus the first molecule is molecule 0.
Total number of input molecules:  1752
```

As indicated in the printed message, the indices corresponding to the molecules that were not correctly processed by *rdkit* are saved to the file **incorrect\_molecules.csv**.
Given that we are using python, these indices start at zero. Thus, be careful when reading these files with other scripting languages, e.g. R.

We will now divide the molecules into a test set (10%), and a training set (90%).

In [7]:

```
stride = int(len(molecules.mols) * 0.9)
training = molecules.mols[0:stride]
test = molecules.mols[stride:len(molecules.mols)]
print len(molecules.mols), len(test), len(training)
```

```
1752 176 1576
```

In [8]:

```
molecules.mols_ids[0:5]
```

Out[8]:

```
['100-03-8', '100-21-0', '100-22-1', '100-37-8', '100-39-0']
```

### A.2 Class to get the substructure IDs:¶

```
bioalerts.FPCalculator.GetDataSetInfo()
```

Next, we are going to extract the substructure information from the molecules contained in the training set, which was derived from the data set previously loaded, namely: *./datasets/Young\_2002.sdf*.

First, initialize the class.

In [9]:

```
training_dataset_info = bioalerts.LoadMolecules.GetDataSetInfo()
```

In [10]:

```
training_dataset_info.extract_substructure_information(radii=[2,3,4],mols=training)
```

We have calculated the substructure information for the molecules in the training set.
In this step, we compute a dictionary of substructures and molecules that will be used later to calculate p.values for
the substructures present in the molecules from the test set.
The keys of the dictionary correspond to substructure IDs, whereas the values correspond to the molecule IDs where a given substructure is present.

Only those substructures of a bond radius within the set of radii especified in the argument *radii* of the method *extract\_substructure\_information* are considered. Therefore, in this example the dictionary of substructures will only contain substructures with a bond radius in the set {2,3,4}.

The length of the substructure dictionary indicates the total number of distinct substructures present in the training set satisfying the bond radii indicated by the user, whereas the number of keys for each dictionary element indicates the number of distinct molecules in which the substructure is present.

For example, the first substructure ID in the dictionary is '4133322759' and appears in molecule 1163:

In [11]:

```
print len(training_dataset_info.substructure_dictionary.keys())
```

```
16092
```

In [12]:

```
training_dataset_info.substructure_dictionary[4133322759]
```

Out[12]:

```
[1163]
```

# B. Calculating p.values for substructures. Categorical bioactivity values¶

Now, we will determine whether the substructures present in the molecules from the test set can be considered as structural alerts or not, on the basis of the information contained in the training set. Only those substructures with a bond radius in the set {2,3,4} will be considered in this example, as this was the only bond radius indicated in the argument **extract\_substructure\_information** when deriving the dictionary of substructures from the training set.

It is important to consider that in this implementation, we do not only focus on the substructural alerts that lead to increased activity. By contrast, we also consider structural alerts that can lead to decreased activity.
Most applications of the derivation of substructural alerts have dealt with the identification of chemical moieties leading to toxicity. The aim being to dismiss compounds harboring them from further testing.

Nevertheless, in the context of hit identification and lead optimization, it can be of practical relevance to also consider those substructures decreasing activity as some substructures can be beneficial for activity against a particular protein A, whereas deleterious for protein B (which can be e.g. an isoenzyme).
Therefore, to attain selectivity towards target A, those substructures deleterious for target B are interesting from a medicinal chemistry standpoint.

Here, the probability for a substructure to be a structural alert is derived from the probability density function of the binomial distribution. To put it in simple words, the question we are asking is (**Figure 1A**):

Which is the probability of having observed (by chance) **m\_S act** active compounds (or more) with a given substructure (**S1**), given that the substructure is present in **n\_s** compounds in a data set of **n** compounds where **m** are active?
A high probability (i.e. p value) indicates that, by chance, it is very likely to have observed **m\_S act** active compounds with substructure **S1**, and thus, substructure **S1** it is not likely to be correlated to activity.
By contrast, a low p value indicates that it is not likely to have obtained **m\_S act** active compounds with substructure **S1** from the underlying binomial distribution defined by the training set. Thus, it is likely that the presence of substructure **S1** is related to compound activity.

# Figure 1¶

To calculate the p values for the substructures in the training set with a bond radius -in this example- in the set {2,3,4}, we first load the bioactivities associated to the molecules from the training set, and which can be found in **molecules.mols**

In [13]:

```
bioactivities = np.genfromtxt('./datasets/Young_2002.dat',
                              dtype='int',
                              skip_header=1,
                              usecols=1)
arr = np.arange(0,len(bioactivities))
mask = np.ones(arr.shape,dtype=bool)
mask[molecules.molserr]=0
bioactivities =  bioactivities[mask]
```

**molecules.molserr** is a list containing the indices for the molecules that could not be properly loaded by rdkit.

We then check that the number of bioactivities and molecules is the same

In [14]:

```
print len(bioactivities)
print len(molecules.mols)
```

```
1752
1752
```

Next, we divide the bioactivities into two sets, corresponding to the **training** and **test** sets defined above.

In [15]:

```
bio_training = bioactivities[0:stride]
bio_test = bioactivities[stride:len(molecules.mols)]
print len(bioactivities), len(bio_test), len(bio_training)
```

```
1752 176 1576
```

We will calculate now the p.values for the substructures present in the molecules from the test set, using the substructure information extracted from the training set.
This is done with the method: **bioalerts.Alerts.calculate\_p\_values()**.
In this example, Alerts\_categorical.calculate\_p\_values().

In the argument **mols**, we provide a list of molecules for which we want to calculate the substructures and calculate their p values.
The method will calculate Morgan fingerprints for these molecules considering as maximal radius the maximal radius
of those provided to the method **training\_dataset\_info.extract\_substructure\_information()** (in this case 4), when we extracted the substructure information for the molecules from the training set.

As a reference set of molecules and substructures, we provide the substructure dictionary calculated for the training set (**substructure\_dictionary=training\_dataset\_info.substructure\_dictionary**). Likewise, we provide the bioactivities for the training set (argument **bioactivities=bio\_training**),
and the molecules ids for the training set (argument **mols\_ids=dataset\_info.mols\_ids[0:stride]**).

We must also provide the threshold for the number of substructures (argument **threshold\_nb\_substructures = 5**),
the p value threshold (argument **threshold\_pvalue = 0.05**), and the threshold for the
substructure frequency (argument **threshold\_frequency = 0.7**).

The explanation for these three arguments is as follows:

1. **threshold\_nb\_substructures**: the threshold for the number of substructures indicates the minimum number of compounds in the training set with a given substructure (**n\_s**) required to proceed the calculation of the p value for that substructure. For instance, if the thresehold is set to 5, and only 4 compounds from the training set present a given substructure *S*, the algorithm will not calculate the p value for *S* when applied to the test set.
2. **thershold\_pvalue**: the p value threshold indicates the level of significance (alpha) to consider a given substructure as structual alert. As said before, the identified strucutural alerts can foster or decrease activity in the biological system under study.
3. **threshold\_frequency**: the threshold for the substructure frequency corresponds to the ratio **m\_S act / n\_s**. Therefore, if there are too few active molecules with a given substructure in the set of molecules (active and inactive) with that substructure, the p values will not be computed. It is important to set a threshold frequency as (quoted from Ahlberg et al.):

"For a substructure to make a statistically significant contribution to activity, it is required that the p-value is below this level. Furthermore, the p-value alone is not sufficient for a substructure to accurately describe an activity. Depending on the skew of the data, the accuracy and p-values required for a significant call will intersect differently. For example, in a 50/50 distribution of positives and negatives, the minimum occurrence for which a substructure can be significant depending on whether its p-value is 5, see Figure 1A. For the majority class of the 70/30 example, the accuracy and p-value are intersected at 50 compounds in the data, see Figure 1B, whereas the minority class is intersected already at three compounds, Figure 1C. This means that, in addition to the level of significance, a lowest level of substructure frequency needs to be imposed on the substructures.

For data where one activity is overrepresented, unbalanced data, it is possible to obtain significant substructures with only two or three occurrences in the data. To avoid this, a lower bound on the number of compounds in which a substructure exists has to be used, denoted minimum occurrence. The minimum occurrence defaults to 5, which derives from the 50/ 50 distributed data and a p-value of 0.05, see Figure 1A."

First, we instatiate the class bioalerts.Alerts.CalculatePvaluesCategorical()

In [16]:

```
Alerts_categorical = bioalerts.Alerts.CalculatePvaluesCategorical(max_radius=4)


Alerts_categorical.calculate_p_values(mols=test,
                                      substructure_dictionary=training_dataset_info.substructure_dictionary,
                                      bioactivities=bio_training,
                                      mols_ids=training_dataset_info.mols_ids[0:stride],
                                      threshold_nb_substructures = 5,
                                      threshold_pvalue = 0.05,
                                      threshold_frequency = 0.7,
                                      Bonferroni=True)
```

```
Number of substructures processed:  4723
Significant substructures:  70 substructures
```

The argument *Bonferroni* indicates wether the Bonferroni correction should be applied to the p values. The Bonferroni correction, which controls the familywise error rate, consists of multiplying the p values by the total number of substructures for which p values were computed (which corresponds to the number of rows of **Alerts\_categorical.output**). Alternatively, one can divide the significance level by the number of computed p values. In that case, the significance level would be then compared to the computed p values to assess the their statistical significance.

In [17]:

```
print len(Alerts_categorical.output)
```

```
70
```

In [18]:

```
float(float(7)/float(7))
```

Out[18]:

```
1.0
```

We see that 355 substructures from the test set can be considered as structural alerts.
For instance:

In [19]:

```
Alerts_categorical.output['Substructure in Molecule'][0]
```

Out[19]:

The results is kept in the *pandas* data.frame **output**.
This data.frame has the following columns:

In [20]:

```
Alerts_categorical.output.columns
```

Out[20]:

```
Index([u'Compound ID', u'Activity label', u'Substructure', u'Substructure in Molecule', u'p_value', u'Compounds with substr.', u'Comp. with substr. active', u'Comp. with substr. inactive'], dtype='object')
```

Beyond all methods of the *pandas* library, the data.frame **Alerts\_categorical.output** can be written to a xlsx file:

In [21]:

```
Alerts_categorical.XlSXOutputWriter(Alerts_categorical.output, 
                                    'test_set_predictions_Young_2002.xlsx')
```

# C. Calculating p.values for substructures. Continuous bioactivity values¶

In the following section, we are going to derive structural alerts from continuous bioactivity data (Figure 1B).
The data set comprises the pIC50 values for 2,311 compounds on cyclooxygenase 2 (COX-2).

In [22]:

```
molecules = bioalerts.LoadMolecules.LoadMolecules("./datasets/COX2.smi",name_field=None)
molecules.ReadMolecules()
```

```
Format of the structures file = SMILES
All molecules in the input file were processed correctly
```

In [23]:

```
cox_bio = np.genfromtxt('./datasets/COX2.bio',skiprows=0)
cox_bio.shape
arr = np.arange(0,len(cox_bio))
mask = np.ones(arr.shape,dtype=bool)
mask[molecules.molserr]=0
cox_bio = cox_bio[mask]
print len(cox_bio)
print len(molecules.mols)
```

```
2311
2311
```

As in the previous section, we divide the data set into a training and a test set.

In [24]:

```
stride = int(len(molecules.mols) * 0.9)
training = molecules.mols[0:stride]
test = molecules.mols[stride:len(molecules.mols)]
print len(molecules.mols), len(test), len(training)
```

```
2311 232 2079
```

In [25]:

```
bio_training = cox_bio[0:stride]
bio_test = cox_bio[stride:len(molecules.mols)]
print len(cox_bio), len(bio_test), len(bio_training)
```

```
2311 232 2079
```

In [26]:

```
training_dataset_info = bioalerts.LoadMolecules.GetDataSetInfo(name_field=None)
training_dataset_info.extract_substructure_information(radii=[2,3,4,5,6],mols=training)
```

In [27]:

```
Alerts_continuous = bioalerts.Alerts.CalculatePvaluesContinuous(radii_ext=[2,3,4,5,6])
```

In [28]:

```
Alerts_continuous.calculate_p_values(mols=test,
                                     substructure_dictionary = training_dataset_info.substructure_dictionary,
                                     bioactivities = bio_training,
                                     mols_ids = molecules.mols_ids[0:stride],
                                     threshold_nb_substructures = 5,
                                     threshold_pvalue = 0.05,
                                     threshold_ratio=0.2)
```

```
number of substructures processed:  16973
```

The arguments of the method **calculate\_p\_values** have the following meanings:

1. **mols:** molecules from which we want to (i) calculate substructures, and (ii) compute p values.
2. **substructure\_dictionary:** reference substructure dictionary derived from the training set.
3. **bioactivities:** bioactivities for the molecules from the training set
4. **mols\_ids:** ids for the molecules from the training set
5. **threshold\_nb\_substructures:** minimum number of compounds with a given substructure required for the algorithm to proceed
6. **threshold\_pvalue:** significance level for the p values.
7. **threshold\_ratio:** minimum ratio for the number of compounds with a given substructure and the total number of compounds in the training set. If the ratio: compounds\_with\_substructure / total\_number\_of\_compounds is smaller than the value input to the argument threshold\_ratio, the algorithm will not consider that substructure.
8. **Bonferroni:** whether the Bonferroni correction should be applied to the p values. The default value is True.

The logic of the algorithm is as follows:

1. Take as input a series of molecules (i.e. training set) and derive a dictionary of substructures.
   Only the substructures with a bond radius within the set of bond radius specified by the user will be considered.
2. For each substructure, S, in the molecules from a test (or external) set, do:

   2.1. If substructure S is in the reference set (derived from the training set), continue

   2.2. If the number of compounds with substructure S in the training set is higher than the value of the argument threshold\_nb\_substructures, continue

   2.3. If the ratio: number\_compounds\_with\_substructure\_S\_training\_set / total\_number\_of\_compounds\_training\_set is higher than the value of the argument threshold\_ratio, continue

   2.4. Compute p.value for substructure S using the Student's t test or the Kolmogorov Smirnov test.

In [29]:

```
len(Alerts_continuous.output)
```

Out[29]:

```
7
```

With the argument values set in this case, we have only idenfied two substructures as significantly implicated in bioactivity

In [30]:

```
Alerts_continuous.output['Substructure in Molecule'][1]
```

Out[30]:

The pandas data.frame **Alerts\_continuous.output** contains the following coloumns:

In [31]:

```
Alerts_continuous.output.columns
```

Out[31]:

```
Index([u'Compound ID', u'Number compounds', u'statistic', u'p_value', u'Diff. distribution means (w - wo)', u'Compounds with substr.', u'Substructure', u'Substructure in Molecule'], dtype='object')
```

Each row corresponds to a substructure. Given that a substructure might appear in more than one compound from the test set, some rows might refer to the same substructure but present in different molecules from the test set. In that case, the columns Compound ID would be different

The columns correspond to:

1. Compound ID: ID of the compound from the test set under consideration
2. Number of compounds: total number of compounds in the training set
3. statistic: the value for the statistic, either (i) the Kolmogorov-Smirnov test statistic, or (i) the Student's t test statistic for the substructure under consideration.
4. p\_value: p value associated to that substructure.
5. Diff. distribution means (w - wo): difference of the mean values between the distributions of bioactivities corresponding to: (i) compounds presenting a given substructure (w), and (ii) compounds not presenting that substructure (wo).
6. Compounds with substr.: number of compounds from the training set presenting the substructure under consideration.
7. Substructure: depiction of the substructure
8. Substructue in Molecule: depiction of the subtructure (in red in the ipython notebook) in the molecule under study (identified by its ID in the column 'Compound ID').

As in the previous case, the results can be written to a .xlsx file:

In [32]:

```
Alerts_continuous.XlSXOutputWriter(Alerts_continuous.output,'test_continuous_COX2.xlsx')
```

# D. Calculating Morgan fingerpints¶

In section D of the tutorial we will see how to calculate hashed and unhashed Morgan fingerprints for a set of molecules, and will train Random Forest models on them.

In [33]:

```
BBB_mols = bioalerts.LoadMolecules.LoadMolecules("./datasets/BBB.smi",name_field=None)
```

In [34]:

```
BBB_mols.ReadMolecules()
```

```
Format of the structures file = SMILES
All molecules in the input file were processed correctly
```

In [35]:

```
BBB_bio = np.genfromtxt('./datasets/BBB.bio',skiprows=0)
print len(BBB_bio)
print len(BBB_mols.mols)
```

```
157
157
```

In [36]:

```
stride = int(len(BBB_mols.mols) * 0.9)
training = BBB_mols.mols[0:stride]
test = BBB_mols.mols[stride:len(BBB_mols.mols)]
print len(BBB_mols.mols), len(test), len(training)

bio_training = BBB_bio[0:stride]
bio_test = BBB_bio[stride:len(BBB_mols.mols)]
print len(BBB_bio), len(bio_test), len(bio_training)
```

```
157 16 141
157 16 141
```

### D.1 Computating HASHED Morgan fingerprints for the TRAINING set:¶

We first initialize the class *bioalerts.FPCalculator.CalculateFPs()*

In [37]:

```
fps_training = bioalerts.FPCalculator.CalculateFPs(radii=[0,1,2],mols=training)
```

In [38]:

```
fps_training.calculate_hashed_fps_binary_quick(nBits=128)
```

In [39]:

```
print fps_training.fps_hashed_binary_quick.shape
```

```
(141, 128)
```

The method *calculate\_hashed\_fps\_binary\_quick()* considers all substructures with a bond radius smaller or equal to
the maximum value of the argument *radii*. In this example, 2.
Thus, in this case this method will consider all substructures with a bond radius of 0, 1 and 2.

In [40]:

```
fps_training.calculate_hashed_fps(nBits=128)
```

In [41]:

```
print fps_training.fps_hashed_binary.shape
print fps_training.fps_hashed_counts.shape
```

```
(141, 128)
(141, 128)
```

The method *calculate\_hashed\_fps()* computes hashed fingerpints in (i) count and (ii) also binary format. The difference
of the method *calculate\_hashed\_fps()* with respect to the method *calculate\_hashed\_fps\_binary\_quick()* is that
the former only consider the substructures with a bond radius in the list input to the argument radii when instantiating the class
*bioalerts.FPCalculator.CalculateFPs()*. In the example above: [1,2].

Therefore, the hashed fingerprints in binary format calculated by these two methods will be identical if the bond radii
especified in the aforesaid argument radii comprise all natural number from 0 to the maximum bond radius.
In this example, this would be correspond to setting the value of the argument radii to [0,1,2].

In [42]:

```
print (fps_training.fps_hashed_binary_quick == fps_training.fps_hashed_binary).all()
```

```
True
```

In [43]:

```
toto = fps_training.fps_hashed_counts
toto[1:10,1:10]
```

Out[43]:

```
array([[0, 0, 1, 1, 0, 0, 0, 1, 0],
       [0, 0, 1, 0, 0, 0, 0, 2, 0],
       [0, 0, 0, 3, 0, 0, 0, 3, 0],
       [0, 0, 0, 0, 0, 1, 0, 3, 0],
       [0, 0, 0, 1, 0, 0, 0, 3, 1],
       [1, 0, 0, 0, 1, 1, 0, 0, 0],
       [0, 0, 1, 0, 0, 1, 0, 1, 0],
       [0, 0, 0, 0, 0, 0, 0, 3, 1],
       [0, 0, 0, 0, 1, 0, 0, 1, 0]])
```

In [44]:

```
fps_training.fps_hashed_binary[1:10,1:10]
```

Out[44]:

```
array([[0, 0, 1, 1, 0, 0, 0, 1, 0],
       [0, 0, 1, 0, 0, 0, 0, 1, 0],
       [0, 0, 0, 1, 0, 0, 0, 1, 0],
       [0, 0, 0, 0, 0, 1, 0, 1, 0],
       [0, 0, 0, 1, 0, 0, 0, 1, 1],
       [1, 0, 0, 0, 1, 1, 0, 0, 0],
       [0, 0, 1, 0, 0, 1, 0, 1, 0],
       [0, 0, 0, 0, 0, 0, 0, 1, 1],
       [0, 0, 0, 0, 1, 0, 0, 1, 0]])
```

In [45]:

```
fps_training.fps_hashed_binary_quick[1:10,1:10]
```

Out[45]:

```
array([[0, 0, 1, 1, 0, 0, 0, 1, 0],
       [0, 0, 1, 0, 0, 0, 0, 1, 0],
       [0, 0, 0, 1, 0, 0, 0, 1, 0],
       [0, 0, 0, 0, 0, 1, 0, 1, 0],
       [0, 0, 0, 1, 0, 0, 0, 1, 1],
       [1, 0, 0, 0, 1, 1, 0, 0, 0],
       [0, 0, 1, 0, 0, 1, 0, 1, 0],
       [0, 0, 0, 0, 0, 0, 0, 1, 1],
       [0, 0, 0, 0, 1, 0, 0, 1, 0]])
```

### D.2 Computating UNHASHED Morgan fingerprints for the TRAINING set:¶

In [46]:

```
fps_training.calculate_unhashed_fps(draw_substructures=True)
```

```
No input set of keys for the substructures. 
Thus, the substructures present in the input molecules will be considered for the calculation of unhashed fingerprints.
```

### D.3 Computating Morgan fingerprints for the TEST set:¶

#### D.3.1 Computating UNHASHED Morgan fingerprints for the test set using a different set of molecules:¶

Since the positions of the substructures in the unhashed fingerprints depend on the training set, the method *calculate\_unhashed\_fps()* allows the computation of unhashed fingerprints for new compounds using a basis defined by the substructures present in the training set. This basis is defined by the keys of the substructure dictionary calculated for the molecules from the training set. This reference substructure dictionary is input to the class *CalculateFPs()* using the argument reference\_substucture\_keys.

This ensures that substructures in new compounds map to the same locations on the fingerprint and allows enhanced model interpretation by noting which exact substructures are deemed important by the learning algorithm.

In [47]:

```
#  Reference molecules
reference_molecules = bioalerts.LoadMolecules.GetDataSetInfo(name_field=None)
```

In [48]:

```
fps_training.substructure_ids
```

Out[48]:

```
array([4272690567, 3416859016, 3818546315, ..., 1182622762, 3218693969,
       3777168895])
```

In [49]:

```
reference_molecules.extract_substructure_information(radii=[0,1,2],mols=training)
```

In [50]:

```
reference_keys = reference_molecules.substructure_dictionary.keys()
print len(reference_keys)
```

```
1456
```

In [51]:

```
fps_test = bioalerts.FPCalculator.CalculateFPs(radii=[0,1,2],
                                         mols=test,
                                         reference_substructure_keys=reference_keys)
```

In [52]:

```
fps_test.calculate_unhashed_fps()
```

In [53]:

```
fps_test.fps_unhashed_counts.shape
```

Out[53]:

```
(16, 1456)
```

In [54]:

```
len(fps_training.columns_unhashed)
```

Out[54]:

```
1456
```

#### D.3.2 Computating HASHED Morgan fingerprints for the test set (e.g. using a different set of molecules):¶

In [55]:

```
fps_test.calculate_hashed_fps(nBits=128)
```

### D.4 Training a Random Forest (RF) model:¶

Two RF models are next trained, using hashed and unhashed Morgan fps, respectively, in count format in both cases.

#### D.4.1 Using hashed fps¶

In [56]:

```
seed = 23
model_hashed_counts = RandomForestRegressor(n_estimators=100,random_state=seed,n_jobs=2)
model_hashed_counts.fit(fps_training.fps_hashed_counts,bio_training)
```

Out[56]:

```
RandomForestRegressor(bootstrap=True, criterion='mse', max_depth=None,
           max_features='auto', max_leaf_nodes=None, min_samples_leaf=1,
           min_samples_split=2, min_weight_fraction_leaf=0.0,
           n_estimators=100, n_jobs=2, oob_score=False, random_state=23,
           verbose=0, warm_start=False)
```

#### D.4.2 Using unhashed fps¶

In [57]:

```
seed = 23
model_UNhashed_counts = RandomForestRegressor(n_estimators=100,random_state=seed,n_jobs=2)
model_UNhashed_counts.fit(fps_training.fps_unhashed_counts,bio_training)
```

Out[57]:

```
RandomForestRegressor(bootstrap=True, criterion='mse', max_depth=None,
           max_features='auto', max_leaf_nodes=None, min_samples_leaf=1,
           min_samples_split=2, min_weight_fraction_leaf=0.0,
           n_estimators=100, n_jobs=2, oob_score=False, random_state=23,
           verbose=0, warm_start=False)
```

### D.5 Predict the bioactivities for the test set:¶

We define two functions for validating our predictions, namely: Pearson's correlation coefficient (R2) and root mean squared errro (RMSE)

In [58]:

```
def Rsquared(pred,true):
    slope, intercept, r_value, p_value, std_err = scipy.stats.linregress(true,pred)
    return r_value**2

def RMSE(pred,true):
    rmse = np.sqrt(mean_squared_error(true,pred))
    return rmse
```

#### D.5.1 Using the model trained on HASHED fps:¶

In [59]:

```
preds_hashed = model_hashed_counts.predict(fps_test.fps_hashed_counts)
```

#### D.5.2 Using the model trained on UNHASHED fps:¶

In [60]:

```
preds_UNhashed = model_UNhashed_counts.predict(fps_test.fps_unhashed_counts)
```

### D.6 Model validation:¶

In [61]:

```
print "RMSE hashed fps: ", RMSE(preds_hashed, bio_test)
print "RMSE UNhashed fps: ", RMSE(preds_UNhashed, bio_test)
```

```
RMSE hashed fps:  0.395223932925
RMSE UNhashed fps:  0.395178569569
```

In [62]:

```
print "R2 hashed fps: ", Rsquared(preds_hashed, bio_test)
print "R2 UNhashed fps: ", Rsquared(preds_UNhashed, bio_test)
```

```
R2 hashed fps:  0.64806507977
R2 UNhashed fps:  0.611070510232
```

In [63]:

```
print "Running time of the whole tutorial in minutes: ", round(time.clock() - initial_time)/60
```

```
Running time of the whole tutorial in minutes:  1.6
```

```
                                  **END OF THE TUTORIAL**

     For any doubts or suggestions, please contact me at isidrolauscher [] gmail [] com
```
